# Genome-scale evidence of the nematode-arthropod clade

**DOI:** 10.1186/gb-2005-6-5-r41

**Published:** 2005-04-28

**Authors:** Hernán Dopazo, Joaquín Dopazo

**Affiliations:** 1 Pharmacogenomics and Comparative Genomics Unit, Bioinformatics Department, Centro de Investigación Príncipe Felipe, Autopista del Saler 16, 46013 Valencia, Spain; 2 Functional Genomics Unit, Bioinformatics Department, Centro de Investigación Príncipe Felipe, Autopista del Saler 16, 46013 Valencia, Spain; 3 Functional Genomics Node, INB, Centro de Investigación Príncipe Felipe, Autopista del Saler 16, 46013 Valencia, Spain

## Abstract

**Background:**

The issue of whether coelomates form a single clade, the Coelomata, or whether all animals that moult an exoskeleton (such as the coelomate arthropods and the pseudocoelomate nematodes) form a distinct clade, the Ecdysozoa, is the most puzzling issue in animal systematics and a major open-ended subject in evolutionary biology. Previous single-gene and genome-scale analyses designed to resolve the issue have produced contradictory results. Here we present the first genome-scale phylogenetic evidence that strongly supports the Ecdysozoa hypothesis.

**Results:**

Through the most extensive phylogenetic analysis carried out to date, the complete genomes of 11 eukaryotic species have been analyzed in order to find homologous sequences derived from 18 human chromosomes. Phylogenetic analysis of datasets showing an increased adjustment to equal evolutionary rates between nematode and arthropod sequences produced a gradual change from support for Coelomata to support for Ecdysozoa. Transition between topologies occurred when fast-evolving sequences of *Caenorhabditis elegans *were removed. When chordate, nematode and arthropod sequences were constrained to fit equal evolutionary rates, the Ecdysozoa topology was statistically accepted whereas Coelomata was rejected.

**Conclusions:**

The reliability of a monophyletic group clustering arthropods and nematodes was unequivocally accepted in datasets where traces of the long-branch attraction effect were removed. This is the first phylogenomic evidence to strongly support the 'moulting clade' hypothesis.

## Abstract 2

The most extensive phylogenetic analysis carried out to date, including 11 complete genomes, is shown to support the Ecdysozoa hypothesis in the open-ended debate of the Coelomata-Ecdysozoa evolutionary problem.

## Background

Understanding the evolution of the great diversity of life is a major goal in biology. Despite decades of effort by systematists, evolutionary relationships between major groups of animals still remain unresolved. The inability to cluster taxa in monophyletic groups was originally due to the lack of morphological synapomorphies among phyla. An alternative solution came from embryology, and animal systematics relied on criteria based on increasing complexity of body plan [1]. Thus, the traditional metazoan phylogeny clusters animals from the simplest basal forms with loose tissue organization (for example, sponges) to those having two germ layers (dipoblastic animals, for example cnidarians), and those developing from three germ layers (triploblastic animals, such as the Bilateria - animals with bilateral symmetry). Bilateral animals were ordered into those lacking a coelom (the acoelomates, such as platyhelminths), those with a false coelom (the pseudocoelomates, such as nematodes), and, finally, those animals with a true coelom (the Coelomata, such as the arthropods and chordates). This comparative developmental theory of animal evolution dominated animal systematics for more than 50 years [2].

Subsequently, molecular systematic studies based on small subunit ribosomal RNA (18S rRNA) sequences began to undermine this scenario [1]. Put briefly, the new animal phylogeny suggested that clades such as acoelomates and pseudocoelomates are artificial systematic groups. Moreover, although the coelomate designation still remains, this clade now contains two new lineages: the lophotrochozoa and the Ecdysozoa [3]. The 'Ecdysozoa hypothesis' postulated that all phyla composed of animals that grow by moulting a cuticular exoskeleton (such as arthropods and nematodes) originate from a common ancestor, thus forming a distinct clade. Thus, under the Ecdysozoa hypothesis arthropods are genetically more closely related to nematodes than to chordates. Under the 'Coelomata hypothesis' of animal evolution, however, arthropods are more closely related to chordates than to nematodes.

At the heart of this systematic debate, a technical discussion emerged surrounding the long-branch attraction effect (LBAE), taxon sampling, and the number of characters used. Subsequent molecular and morphological studies have been carried out, but the controversy remains unresolved and is presented as a multifurcation [4]. Although the use of different single-gene sequences supported the Ecdysozoa hypothesis [5-11], the analysis of dozens to hundreds of concatenated sequences supported the Coelomata clade [12-15]. Indeed, with an element of caution, we favored the Coelomata hypothesis in a previous whole-genome study designed to determine the number of characters needed to obtain a reliable topology [16]. The gene-based Ecdysozoa versus genome-scale Coelomata alternative hypotheses were recently challenged by two phylogenomics studies that partly supported the Ecdysozoa clade [17] and a paraphyletic Coelomata group [18]. Although it is generally accepted that phylogenetic analysis of whole genomes has begun to supplement (and in some cases improve on) phylogenetic studies previously carried out with one or a few genes [19], all genome-wide phylogenetic studies have failed to support the proposed new animal phylogeny.

Here we present the first phylogenomic evidence that strongly supports the Ecdysozoa hypothesis and at the same time demonstrates that the LBAE biases the position of *Caenorhabditis elegans *in the phylogenetic tree. We show that by using a large number of characters and choosing a phylogenetic weighted scheme of outgroups to test the constancy of evolutionary rates, the new animal phylogeny can be statistically supported. Moreover, we show that both the Coelomata and the Ecdysozoa hypotheses can be supported with the highest statistical confidence when genomic datasets are ordered according to a gradually increased adjustment to equal evolutionary rates between *C. elegans *and *Drosophila melanogaster *sequences. In between, neither Ecdysozoa nor Coelomata were sufficiently supported. To our knowledge, this is the most extensive phylogenomic analysis carried out to date in the number of characters and the number of eukaryotic species involved.

## Results

### Dataset properties

**Figure 1:**
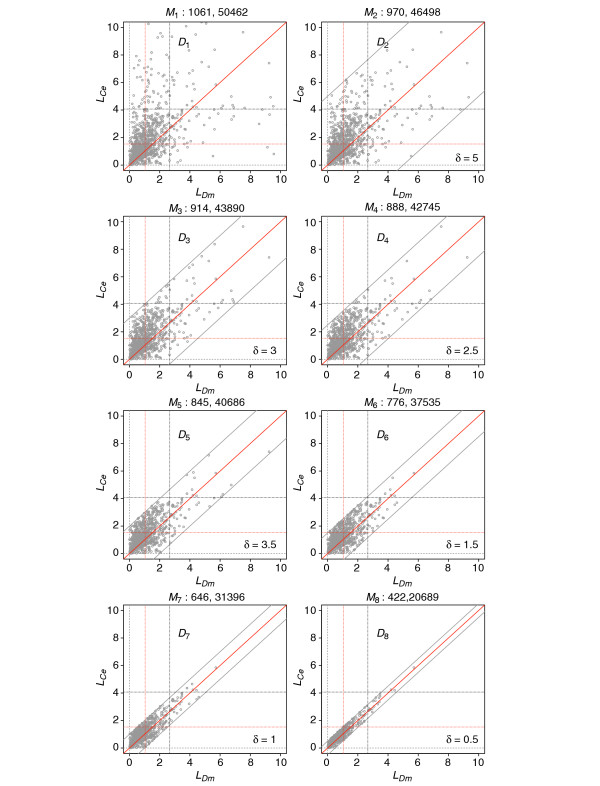
Description of the dataset. *D*_*i *_datasets are arranged according to a gradual decrease in the parameter δ. δ controls the inclusion of each homologous exon sequence in the dataset by defining margins above and below (*y *= *x *± δ) a diagonal line (*y *= *x*) that constrains clock-like behavior in the evolution of *C. elegans *and *D. melanogaster *sequences. *L*_*Ce *_and *L*_*Dm *_are the respective relative branch lengths of *C. elegans *and *D. melanogaster *using *H. sapiens *as reference. Comma-separated values represent the number of homologous sequences and characters aligned in the *M*_*i *_concatenated matrix. *D*_*i *_contains all the sequences without any constraint of evolutionary rates. Dotted black and red lines represent mean ,  and median values, respectively.

Sequences homologous to human exon sequences were derived from filtering tblastn search results on 11 complete eukaryotic genomes. Because the most-criticized issue in resolving the Ecdysozoa-Coelomata problem seems to be the LBAE produced by the nematode species, we decided to rearrange homologous sequences in a series of nested datasets that gradually reduced LBAE. Aligned homologous sequences were arranged in eight datasets (*D*_*i*_) and concatenated in their corresponding matrices (*M*_*i*_) (see Materials and methods), such that as suffix *i *increases, datasets and matrices comprise a smaller number of homologous sequences showing more similar relative branch lengths (RBL) between *C. elegans *(*L*_*Ce*_) and *D. melanogaster *(*L*_*Dm*_) (Figure 1). RBL are relative human distances.

**Figure 2:**
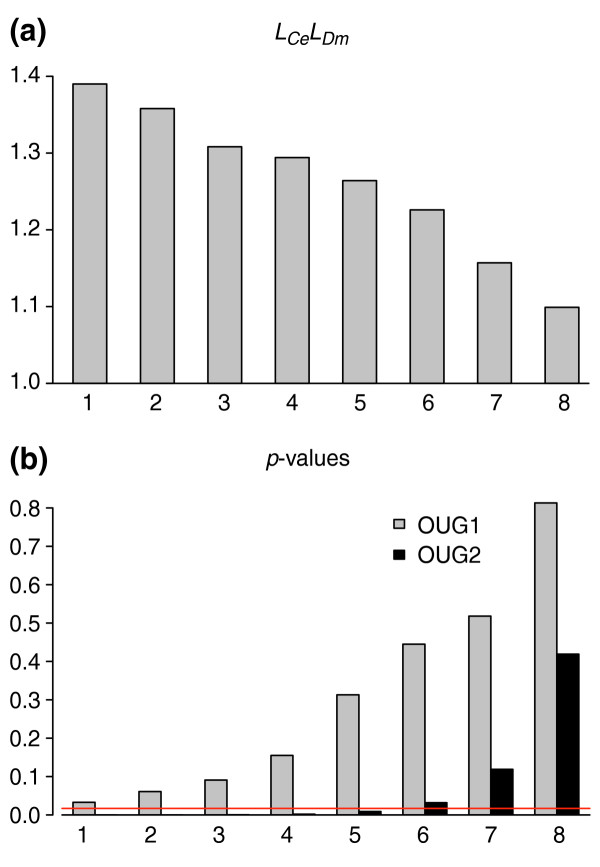
Relative rate test. **(a) **Relative *C. elegans *branch lengths derived from each one of the eight *M*_*i *_matrices. Maximum likelihood estimates are expressed as relative distance units of *D. melanogaster*. **(b) **Relative rate test probability values evaluated at the 5% level family-wise (red line 1.7%). OUG1, *S. cerevisae*; OUG2, phylogenetic weighted scheme using *S. cerevisae*, *A. thaliana*, *O. sativa *and *P. falciparum *as outgroup species.

To quantify the effect on the RBL of *C. elegans *of concatenating alternative homologous sequences, maximum likelihood (ML) estimates of branch length were obtained using the star-like unrooted tree transformation for each dataset (see Materials and methods). Figure 2a shows that the RBL of *C. elegans *over *D. melanogaster *decreased by approximately 30% continuously from dataset *D*_1 _to *D*_8_. To test whether the gradual decrease in *C. elegans *branch length was enough to produce statistical confidence on equal evolutionary rates between the nematode and the arthropod sequences, relative rate tests using two outgroup schemes were assayed on concatenated sequences (see Materials and methods). Figure 2b shows that using *Saccharomyces cerevisae *as the unique outgroup species (OUG1), all the individual tests on the eight matrices failed to detect statistical deviations (at the 5% level family-wise) between sequences. Only when the phylogenetically weighted scheme of outgroup species (OUG2) was used did the relative rate test detect significant deviation of clock behavior from *D*_1 _to *D*_5 _datasets. We are therefore confident that the arthropod and nematode concatenated sequences of the *M*_6_, *M*_7_, and *M*_8 _matrices meet the desired clock-like conditions to test the Coelomata and Ecdysozoa hypotheses and exclude any artifacts derived from a possible LBAE. This result supports previous work suggesting that the genetic distance between ingroup and outgroup modifies the power of the relative rate test [20].

To test whether concatenated matrices carry sufficient phylogenetic signal, the ML mapping method was used. The compound posterior probability point (*P*) for all the possible quartets of each *M*_*i *_matrix could be placed, with almost equivalent values (approximately 33%), inside the corner areas of the equilateral triangle probability surface (see Additional data file 1). Thus, concatenated matrices derived from selecting a different number of homologous sequences contained sufficient phylogenetic signal to represent topologies as strictly bifurcating trees. Finally, using the Akaike information criterion (AIC) [21], the statistical test of the best-fit model of sequence evolution for each dataset was selected from six different alternatives (see Materials and methods). As all the models are not nested and share the same number of parameters, the best one was that with the greatest log likelihood result. The WAG amino-acid replacement matrix [22] adjusted for frequencies (+*F*), rate heterogeneity (+Γ) and invariable sites (+*I*) was the best evolutionary model chosen for all the datasets. Moreover, model-fit-data values followed the same inequality independently of the dataset (WAG [22] > VT [23] > BLOSUM62 [24] > JTT [25] > PAM [26] > mtREV24 [27]), suggesting that the best models were those that consider more distantly related amino-acid sequences.

### The clade Coelomata disappears under clock conditions

**Figure 3:**
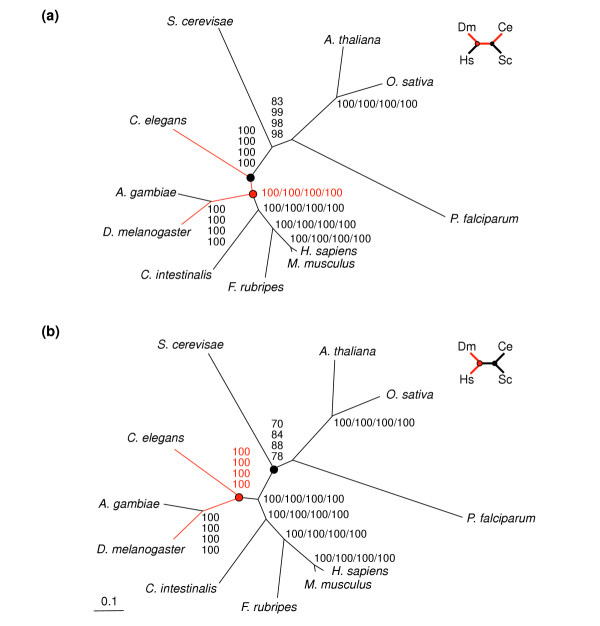
Phylogenetic trees. Trees derived from *M*_1 _and *M*_8 _datasets, respectively support (a) the Coelomata and (b) the Ecdysozoa hypothesis. From left to right or top to bottom, values besides nodes show the maximum likelihood reliability values of the quartet-puzzling tree and bootstrap values using maximum likelihood, least squares, and neighbor-joining methods, respectively. Values in red show the support for (a) Coelomata and (b) Ecdysozoa nodes. Red branches display distances between *C. elegans *and *D. melanogaster*. Smaller trees are minimal representations of both hypothesis.

**Figure 4:**
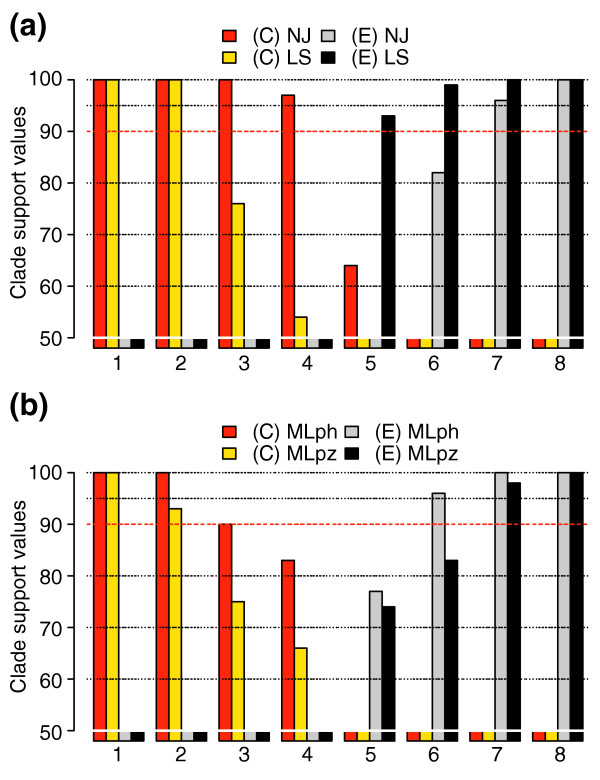
Bootstrap and reliability support for alternative topologies. Bootstrap and reliability support (50% majority consensus rule) for Coelomata (C) and Ecdysozoa (E) hypotheses derived from each one of the eight *M*_*i *_matrices. **(a) **Distance methods. LS, least squares; NJ, neighbor joining. **(b) **Maximum likelihood, using PHYLIP (ph) and PUZZLE (pz). Highly supported trees were considered those with values above 90% (dotted red line).

Distance and ML phylogenetic methods were used on all the datasets (see Materials and methods). Figure 3 shows phylogenetic reconstructions and statistical support for the two extreme conditions of the nested datasets. Whereas the *M*_1 _matrix supported the Coelomata tree with the highest statistical confidence, *M*_8 _showed the same result for the Ecdysozoa tree. Thus, by decreasing the RBL of *C. elegans*, the statistical support switched from the Coelomata to the Ecdysozoa hypothesis. Figure 4 shows that, whichever phylogenetic method was used, *C. elegans *bootstrap support between datasets and topologies changed in agreement with the gradual RBL decrement. Specifically, using *M*_1 _and *M*_8 _(the matrices showing the most extreme evolutionary rate conditions for *C. elegans *and *D. melanogaster *sequences - from a clock-absent to the most adjusted behavior), the statistical support moved from Coelomata to Ecdysozoa. The same occurred with *M*_2 _and *M*_7_. Alternatively, using *M*_3 _and *M*_6_, only one of the two distance and ML methods (Figure 4a,b) provided sufficient support (90% or more) to the hypothesis. Finally, using *M*_4 _and *M*_5_, only one distance method supported Coelomata and Ecdysozoa with confidence. Given that datasets differed principally in the RBL of *C. elegans *over *D. melanogaster*, the gradual change in topology strongly favors an LBAE between *C. elegans *and the more basal species. To test whether a paired-sites test [28] supports the bootstrap conclusions, Shimodaira-Hasegawa (SH) and expected-likelihood weight (ELW) tests were evaluated on the datasets (see Materials and methods).

**Figure 5:**
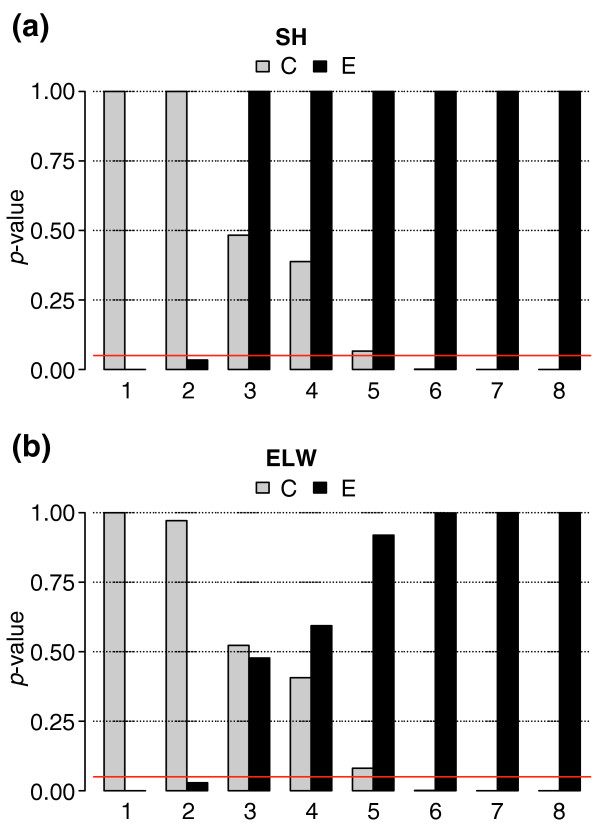
Paired-sites tests. *p*-values inferred from paired-sites tests considering Coelomata (C) and Ecdysozoa (E) hypotheses at the 5% level (red line) for all the datasets. (**a**) Shimodaira-Hasegawa test (SH); (**b**) expected-likelihood weight method (ELW).

Figure 5 shows the assessment of paired-sites tests for the two competing trees on all the datasets. Paired-sites tests supporting topologies (*p *> 0.05) changed almost gradually on datasets. Figure 5a and 5b show that the SH test is more conservative than the ELW [29]. Using matrices *M*_1 _and *M*_2_, both tests strongly rejected the Ecdysozoa hypothesis, whereas *M*_6_, *M*_7_, and *M*_8 _rejected the Coelomata tree. Interestingly, datasets between them did not reject any topology with sufficient statistical evidence. We can conclude that by decreasing the RBL of *C. elegans *over *D. melanogaster *by around 13% (Figure 2a) the LBAE favoring the Coelomata hypothesis disappears and we can confirm that under strict conditions of clock-like behavior, the Coelomata hypothesis was strongly rejected by paired-sites tests and bootstrap support.

To test if the shortness of the evolutionary distances between *C. elegans *and *D. melanogaster *resulting from the above filtering method biased topology over the common ancestry of arthropods and nematodes, we searched for chordate, arthropod, and nematode sequences showing clock-like behavior between them. To increase the probability of finding sequences to fit the criteria, we focused on sequences from the most closely related chordate to the molting species, that is, the ascidian *Ciona intestinalis*. Only 14 exon sequences met the above criteria. A relative rate test showed that the probability of a perfect clock-like behavior was *p *= 0.515 for *C. elegans *and *D. melanogaster*, *p *= 0.308 for *C. intestinalis *and *D. melanogaster *and *p *= 0.712 for *C. intestinalis *and *C. elegans*. The ML mapping method showed that the concatenation of all the 810 characters carried sufficient phylogenetic signal in the matrix to represent a strictly bifurcating tree (see Additional data file 2). Despite the reduced number of characters, phylogenetic analysis showed significant support for the Ecdysozoa hypothesis. Using distance and ML methods, bootstrap values reached 97%. Moreover, the Ecdysozoa hypothesis was accepted with a probability of *p *= 1.00 and *p *= 0.997 when SH and ELW paired-sites tests, respectively, were performed. Conversely, the Coelomata hypothesis was rejected at *p *= 0.006 and *p *= 0.0023, respectively.

### The clade Coelomata disappears by removing fast-evolving sequences of *C. elegans*

**Figure 6:**
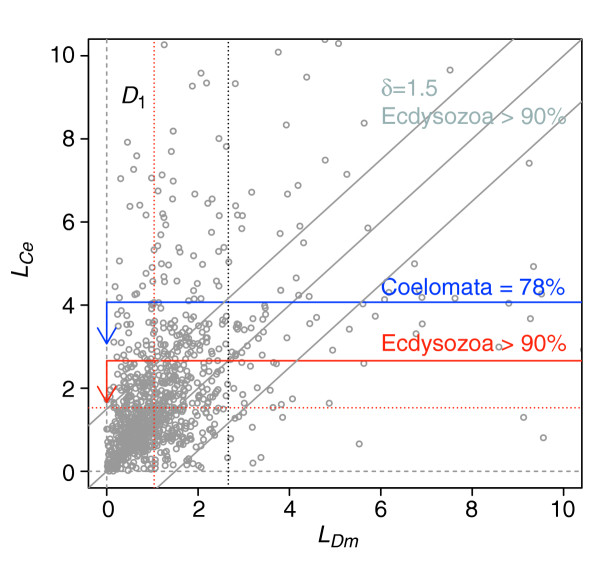
Removing fast-evolving sequences. Exon sequences of *C. elegans *showing *L*_*Ce *_≥  = 4.06 represent 15% of the total exon. When these faster exons were removed (above blue line), support for the Coelomata topology was reduced from the original 100% to 85%. Furthermore, when 28% of the faster exons were deleted (red line), Ecdysozoa is recovered with 90% statistical support. This suggests that LBAE is the main problem in obtaining the Ecdysozoa tree. Blue line,  = 4.06; red line,  = 2.66.

In order to discard a probable biased selection of exon sequences favoring the Ecdysozoa hypothesis, two additional matrices were built by removing from the original dataset (*D*_1_) the exons in which the *C. elegans *sequences evolved at a faster rate. Figure 6 shows that by removing the fastest 15% of total exon sequences the reliability of the Coelomata hypothesis is reduced from 100% to 78%. Moreover, when the fastest 30% of all exons were removed, the topology changes to Ecdysozoa with 90% confidence level. The change in topology in parallel with the reduction of the *C. elegans *branch length points to the LBAE as the main obstacle to obtaining the true phylogenetic relationship between chordates, arthropods and nematodes. We conclude that the Ecdysozoa hypothesis does not depend on adjusting a particular set of homologous exon sequences to clock-like behavior.

## Discussion

There are many reasons why the Coelomata-Ecdysozoa problem should be considered the most puzzling problem in animal systematics and a major open-ended subject in evolutionary biology. The monophyly of the Ecdysozoa group, strongly championed by the evo-devo community [30], was originally deduced, and continually recovered, through the analysis of different single-gene sequences [3,5,6,8-11], sometimes in combination with morphological characters [7]. There is need for caution, however, as previous studies had shown that individual genes are not sufficient to estimate the correct genome phylogeny [19,31]. Furthermore, the reliability of some of the phylogenetic markers used to derive Ecdysozoa has been seriously questioned [32,33]. Those that consider the Ecdysozoa hypothesis as more plausible insist that the Coelomata topology is an artifact of LBAE, derived from the fact that nematode genomes, particularly that of *C. elegans*, evolve at higher rates [3], and are consequently displaced to a more basal position.

On the other hand, as phylogenetic reconstruction assumes that sampled data are representative of the whole genome from which they are drawn [34], there is increasing agreement to consider genome-scale analysis more accurate than single-gene analysis when deciding between conflicting topologies [19,31]. Conflict derives from the fact that all previous genome-wide phylogenetic attempts to test the hypothesis have failed to confirm the 'moulting group' - the Ecdysozoa - as a clade. All phylogenomic analyses carried out to date favor the Coelomata hypothesis with the highest statistical support [12-16]. Furthermore, the Coelomata tree has shown to be robust to criticism deriving from LBAE [12,14-16] and nematode species inclusion [14]. Those that consider the Coelomata hypothesis to be more appropriate insist that longer sequences, rather than extensive taxon sampling [35], will more effectively improve the accuracy of phylogenetic inference [14,15,36,37], and emphasize that an inevitable trade-off exists between the number of characters and the number of species used in the study [15].

We show here that by using the fast-evolving nematode *C. elegans *the Ecdysozoa can be recovered using genome-scale phylogenetic analysis. Our analysis has been performed over the largest number of eukaryotic genomes and over the largest number of amino-acid residues ever used to test the hypothesis. The major differences from previous genomic approaches are threefold. First, we used a large number of short conserved sequences (around 50 amino acids long) derived from human homologous exon sequences. Only exon sequences derived from eight genes, out of a total of around 100 analyzed by Blair *et al*. [14], were used in our analysis. The remaining genes contained in the 18 human chromosomes did not pass the BLAST filters applied in the analysis. Second, we arranged the dataset such that the sequences, including those evolving faster or slower, were included if they met the condition of equal rate of change between two (*C. elegans *and *D. melanogaster*) or three species (*C. intestinalis*, *D. melanogaster *and *C. elegans*). Third, we used a large number of characters (amino-acid residues) and a weighted distant outgroup species to enhance the power of the relative rate test [20].

As discussed in our previous paper [16], by including or excluding certain human homologous exon sequences, we reduced the problem of LBAE and added a probable bias favoring Coelomata. The present work confirms that this bias exists. The concatenation and the posterior phylogenetic analysis of the sequences shared by the eukaryotes used in this analysis provide a viable solution to the ancestor-descendant relationships of animal species once the LBAE is removed.

## Conclusions

Acceptance of the new animal phylogeny and the Ecdysozoa hypothesis would provide a new scheme to understand the Cambrian explosion [38,39] and the origin of metazoan body plans [9,30] and consequently would set a new phylogenetic framework for comparative genomics [40]. We have shown how phylogenetic reconstruction based on whole-genome sequences has the potential to solve one of the most controversial hypotheses in animal evolution: the reliability of the Ecdysozoa clade.

## Materials and methods

### Dataset collection

Complete genome sequences from *Plasmodium falciparum *[41], *Arabidopsis thaliana *[42], *Oryza sativa *[43], *Saccharomyces cerevisae *[44], *Caenorhabditis elegans *[45], *Anopheles gambiae *[46], *Drosophila melanogaster *[47], *Ciona intestinalis *[48], *Fugu rubripes *[49], *Mus musculus *[50] and *Homo sapiens *[51] were downloaded and formatted to run local BLAST [52]. Amino-acid sequences corresponding to all the gene exons in a sample of 18 human chromosome including 6-18, 20-22, X and Y (approximately 14,000 genes and 140,000 exons), were obtained from the Ensembl database project [53]. Human paralogous exons were excluded by running local blastp [52] on a human exon database built *ad hoc*. Only the best of those sequences, with more than a single hit with a fraction of aligned and conserved amino-acid sequence ≥ 95% and ≥ 90% respectively, were retained to find homologous sequences in the other eukaryotic species (threshold values based on a previous human paralogous study [54]). We used tblastn [52] that searches a query amino-acid sequence on the six translation frames of the target sequence to search for homology in the complete genome databases of the species mentioned above. Exons less than 22 amino acids were removed from the analysis. Each best hit of tblastn was filtered by means of a threshold e-value (≤ 1*e*-03) and a threshold proportion of the query over the subject sequence length (≥ 75%). Only those exons that pass through all the species filter conditions were selected as the final dataset of human exon homologous sequences. All the exon homologous sequences were aligned using Clustal W [55] with default parameters. The total number of homologous sequences, derived from 18 human chromosomes, corresponds to 1,192 exons selected from 610 known genes, adding up to more than 55,500 amino-acid characters.

To arrange homologous sequences in different datasets, pairwise distances between sequences were extracted using the PROTDIST program (Kimura option) of the PHYLIP package [56]. Distances between *C. elegans*, *D. melanogaster *and *H. sapiens *were transformed into branch lengths in a star-like unrooted tree (*l*_*a *_= (*d*_*ab *_+ *d*_*ac *_- *d*_*bc*_)/2, where *l*_*a *_is the length of the branch leading to *a *and *d*_*ab*_, *d*_*ac*_, *d*_*bc *_are the distances between *a *and *b*, *a *and *c*, and *b *and *c*, respectively). It is important to emphasize that we are not considering that the phylogenetic relationships of *C. elegans*, *D. melanogaster *and *H. sapiens *is a star topology. We used this exact equation for determining the branch lengths of the three species, because the unique way to arrange three species in a phylogenetic tree is a star topology. We consider *C. elegans*, *D. melanogaster *and *H. sapiens *to be members of the ingroup and *P. falciparum*, *A. thaliana*, *O. sativa *and *S. cerevisae *as the outgroup species at the moment to root the phylogenetic tree. Homologous exon sequences were arranged in eight datasets according to their pertinence to more inclusive areas surrounding the straight line representing identical relative branch lengths (RBLs) of *C. elegans *(*L*_*Ce *_= *l*_*Ce*_/*l*_*Hs*_) and *D. melanogaster *(*L*_*Dm *_= *l*_*Dm*_/*l*_*Hs*_). The *D*_*i *_dataset clusters all the homologous exon alignments where *L*_*Dm *_- δ_*i *_≤ *L*_*Ce *_≤ *L*_*Dm *_+ δ_*i*_, where *i *is an integer ranging from 2 to 7 and δ_*i *_= 5.0, 3.0,2.5,2.0,15,1.0,0.5. The *D*_1 _dataset contains all the exon homologous sequences without the constraints of evolutionary rates. Exons with negative or undefined normalized distances (*l*_*Hs *_= 0) were excluded from the analysis. All the aligned homologous exon sequences of the *D*_*i *_dataset were concatenated in the *M*_*i *_matrix. Three additional matrices were derived from *D*_1_: two by removing exons containing *L*_*Ce *_≥  and *L*_*Ce *_≥ , and the last one by adjusting the sequences of *C. intestinalis*, *D. melanogaster *and *C. elegans *to clock-like behavior.

### Phylogenetic methods

The relative rate test was performed at the 5% statistical level by means of the RRTree program [57] using outgroups with one (*S. cerevisae*; OUG1) or more species (*S. cerevisae*, *A. thaliana*, *O. sativa *and *P. falciparum*; OUG2). In the latter case, an explicit weighted phylogenetic scheme was chosen (1/2 *S. cerevisae*, ((1/8 *A. thaliana*, 1/8 *O. sativa*), 1/4 *P. falciparum*)). Given that three ingroups were set for all analyses (the chordates *H. sapiens*, *M. musculus*, *F. rubripes*, and *C. intestinalis*; the arthropods *Anopheles gambiae *and *Drosophila melanogaster*; and the nematode *C. elegans*), the threshold value was corrected for multiple testing to 5/3 = 1.7%. TREE-PUZZLE [58] was used to evaluate six alternative evolutionary models adjusted for frequencies (+*F*), site rate variation (+Γ distribution with two rates) and a proportion of invariable sites (+*I*), to estimate the amount of evolutionary information of datasets by the likelihood-mapping method [59], to derive the maximum likelihood (ML) trees using the quartet-puzzling algorithm, to set the ML pairwise sequence distances, and to test alternative topologies using SH [60] and ELW [29] tests. The PROML (JTT+f) program of the PHYLIP package [56] was used to estimate ML trees derived from the stepwise addition algorithm. Distance methods of phylogenetic reconstruction were performed using PROTDIST (JTT, Kimura options), NEIGHBOR (neighbor-joining (NJ) [61]) and least squares (LS) [62] algorithms, and CONSENSE (50% majority-consensus rule option) programs on 100 bootstrap replications using PHYLIP.

## Additional data files

The following additional data files are available with the online version of this paper. Additional data file 1 contains a figure showing ML puzzle mapping of the *M*_*i *_matrices. Additional data file 2 contains a figure showing ML puzzle mapping of the matrix derived from chordate, arthropod and nematode sequences showing clock-like behavior. Additional data file 3 contains the matrices.

## Supplementary Material

Additional File 1 **ML puzzle mapping of the *M*_*i *_matrices. **Maximum likelihood mapping results for each one of the *M*_*i *_concatenated matrices. From the first row and from left to right, M1 to M2 until the fourth row, M7 to M8. Click here for file

Additional File 2 **ML puzzle mapping of the matrix derived from chordate, arthropod and nematode sequences showing clock-like behavior.** ML mapping of the concatenated matrix derived from constraining sequences to 3 clocks-like behavior. Click here for file

Additional File 3 **Matrices. **The full set of matrices (phylip format) used in the phylogenetic analyzes. Click here for file
