# Supplementary figures and images for: Genome-scale evidence of the nematode-arthropod clade
Source: Genome Biol. 2005 Apr 28;6(5):R41. doi: 10.1186/gb-2005-6-5-r41 (PMC1175953; doi:10.1186/gb-2005-6-5-r41)

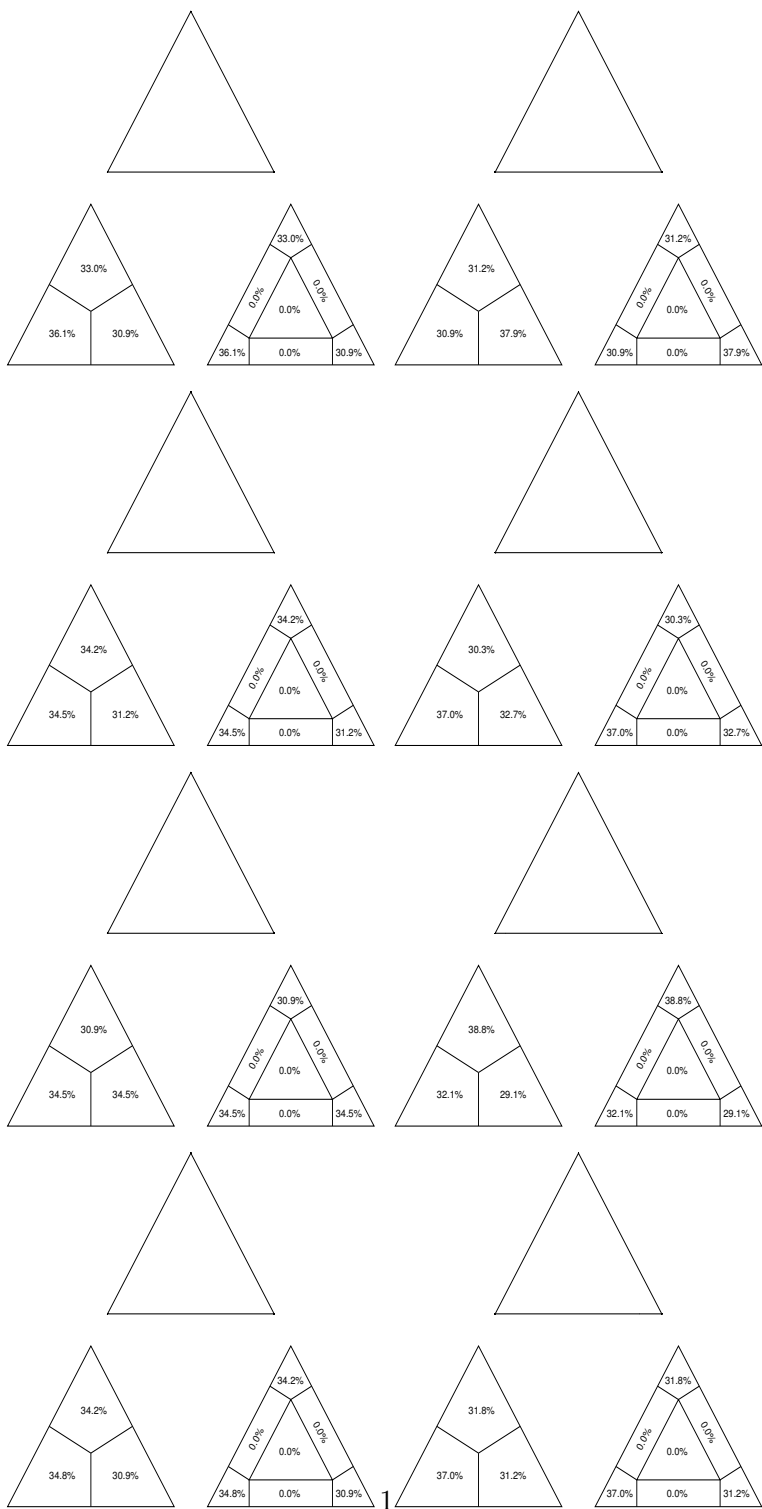

Supplement: Additional File 1 — ML puzzle mapping of the Mi matrices. Maximum likelihood mapping results for each one of the Mi concatenated matrices. From the first row and from left to right, M1 to M2 until the fourth row, M7 to M8. [file gb-2005-6-5-r41-S1.pdf]

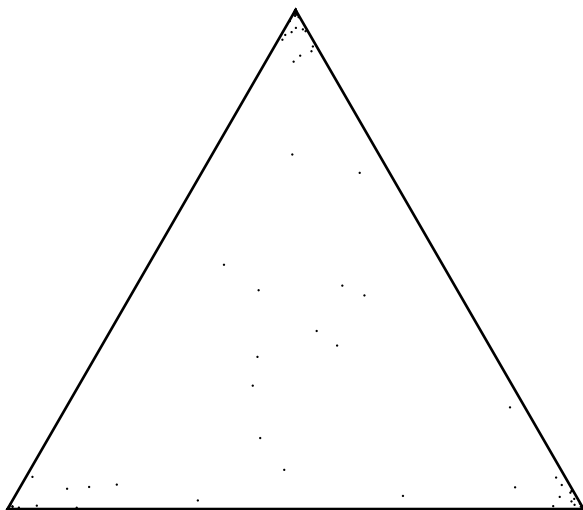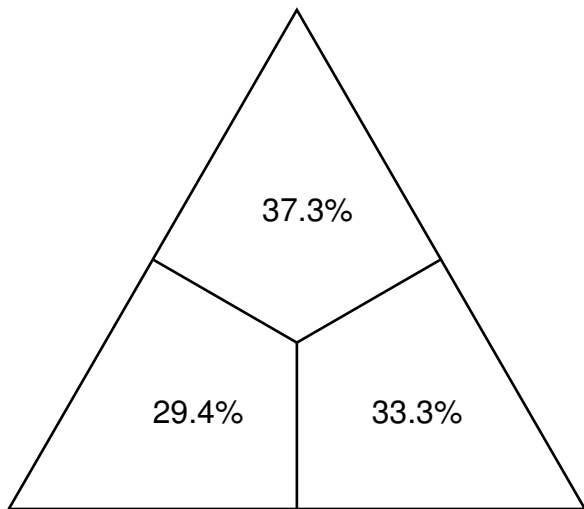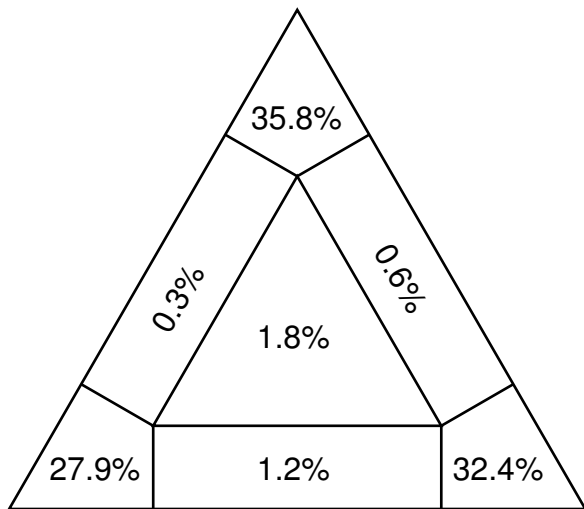

Supplement: Additional File 2 — ML puzzle mapping of the matrix derived from chordate, arthropod and nematode sequences showing clock-like behavior. ML mapping of the concatenated matrix derived from constraining sequences to 3 clocks-like behavior. [file gb-2005-6-5-r41-S2.pdf]
